# Performance Analysis of Ionospheric Scintillation Effect on P-Band Sliding Spotlight SAR System

**DOI:** 10.3390/s19092161

**Published:** 2019-05-09

**Authors:** Lei Yu, Yongsheng Zhang, Qilei Zhang, Yifei Ji, Zhen Dong

**Affiliations:** College of Electronic Science and Technology, National University of Defense Technology, Changsha 410073, China; yulei17@nudt.edu.cn (L.Y.); zhangqilei@nudt.edu.cn (Q.Z.); jyfnudt@163.com (Y.J.); dongzhen@nudt.edu.cn (Z.D.)

**Keywords:** ionosphere, P-band, reverse back-projection (ReBP), synthetic aperture radar (SAR), sliding spotlight, scintillation

## Abstract

The space-borne P-band synthetic aperture radar (SAR) maintains excellent penetration capability. However, the low carrier frequency restricts its imaging resolution. The sliding spotlight mode provides an operational solution to meet the requirement of high imaging resolution in P-band SAR design. Unfortunately, the space-borne P-band SAR will be inevitably deteriorated by the ionospheric scintillation. Compared with the stripmap mode, the sliding spotlight SAR will suffer more degradation when operating in the scintillation active regions due to its long integration time and complex imaging geometry. In this paper, both the imaging performance and scintillation effect for P-band sliding spotlight mode are studied. The theoretical analysis of scintillation effect is performed based on a refined model of the two-frequency and two-position coherence function (TFTPCF). A novel scintillation simulator based on the reverse back-projection (ReBP) algorithm is proposed to generate the SAR raw data for sliding spotlight mode. The proposed scintillation simulator can also be applied to predict the scintillation effect for other multi-mode SAR systems such as terrain observation by progressive scans (TOPS) and ScanSAR. Finally, a group of simulations are carried out to validate the theoretical analysis.

## 1. Introduction

It is widely known that synthetic aperture radar (SAR) system working at P-band shows its superiority in penetrating the forest foliage and the ground surface, which will have an extensive application prospect in biomass measurement and geological observation [1,2,3]. Therefore, there has been an upward trend of developing P-band SAR, for example the BIOMASS mission [4,5]. Despite the remarkable advantages, there are two main drawbacks existing in the space-borne P-band SAR systems. One is the severe susceptivity of the ionospheric impact [6,7,8,9], especially for the equatorial scintillation effect. The other is the limitation of azimuth resolution which is restricted by the low central frequency.

To elevate the azimuth resolution and maintain adequate imaging swath, the sliding spotlight mode has been used in many SAR systems such as TerraSAR-X and PAMIR [10,11,12,13]. The sliding spotlight mode controls the scanning velocity of beam footprint by steering the antenna, thus obtaining longer integration time than stripmap mode and larger scene than spotlight mode. Consequently, the sliding spotlight mode is a practical way for P-band high-resolution SAR system. However, little literature has been proposed to evaluate the ionospheric effect for P-band sliding spotlight SAR system.

The intensive solar radiation results in the ionization of ionospheric molecule. Varying from the scale of spatial distribution, the ionosphere is typically categorized into the background ionosphere (larger than 10 km) and ionospheric irregularities (less than 10 km) [8,14]. The background ionospheric effect can be mitigated using the split-spectrum method or using the ionospheric prior knowledge acquired from the global navigation satellite system (GNSS)/BeiDou system [15,16,17,18]. In recent papers, the multi-squint (MS) interferometry methodology is proposed [5], which provides a new ionospheric mitigation approach for SAR system with limited bandwidth. The scintillation effect is caused by small scale ionospheric turbulent irregularities, which typically occurs after the sunset in the equatorial and polar regions [14]. The strong scintillation effect, which usually shown as streaks in SAR images, has been extensively reported by the Advanced Land Observing Satellite (ALOS)/the Phased Array-type L-band Synthetic Aperture Radar (PALSAR) system. The scintillation turbulence on both amplitude and phase will introduce serious spatial and frequency decorrelation within the SAR integration time and further distort the imaging performance. The former research usually focuses on the analysis of the scintillation effect for stripmap SAR systems [19,20,21,22,23,24,25,26]. The generalized ambiguity function (GAF) proposed by Ishimaru [19] in 1999, provides a comprehensive model to evaluate the degradation of signal coherence introduced by the ionospheric effect. Based on the GAF model, Li et al. [20] introduced the two-frequency and two-position coherence function (TFTPCF) into the traditional GAF model to evaluate the scintillation-induced signal decorrelation. The analysis of anisotropic irregularity is performed by C. Wang [23] and a statistical evaluation of L-band equatorial scintillation is carried out by Meyer [24]. The SAR scintillation simulator (SAR-SS) is studied by Carrano [27] based on the phase screen theory for predicting the scintillation effect on the L-band SAR. In Carrano’s work, the inverse range-Doppler algorithm (RDA) is applied to generate the unaffected SAR signal. However, it cannot actually simulate the observation geometry of SAR system, thus it is not suitable to reconstruct the SAR raw data for the sliding spotlight SAR system.

The former research builds the foundation of our work. However, these achievements only take the stripmap mode into consideration and further research still needs to be accomplished by considering the sliding spotlight observation geometry. Compared with the stripmap mode, the P-band sliding spotlight SAR system has an ultra-long integration time and more complicated observation geometry which means a longer exposure time and a longer ionospheric penetration length (IPL) in scintillation active regions. Due to the beam scanning, the incident angle of beam center, which is an important parameter in scintillation simulations, varies within the acquisition time. All these characteristics will make the scintillation effect on sliding spotlight mode show different patterns.

In this paper, we firstly introduce the observation geometry of sliding spotlight SAR system in Section 2. Then, in Section 3, the theoretical analysis of scintillation effect is performed based on the refined GAF and TFTPCF model. The comparisons between sliding spotlight mode and stripmap mode are presented. In Section 4, a novel SAR-SS is proposed by considering the beam scanning of sliding spotlight mode. The reverse back-projection (ReBP) algorithm [28] is applied to generate the sliding spotlight mode SAR raw data. Finally, the simulations are performed on both point target and extended target to demonstrate the scintillation-induced imaging distortion. A group of 500-time Monte-Carlo simulations are carried out to validate the theoretical analysis.

## 2. The Observation Geometry of Sliding Spotlight SAR System

**Figure 1:**
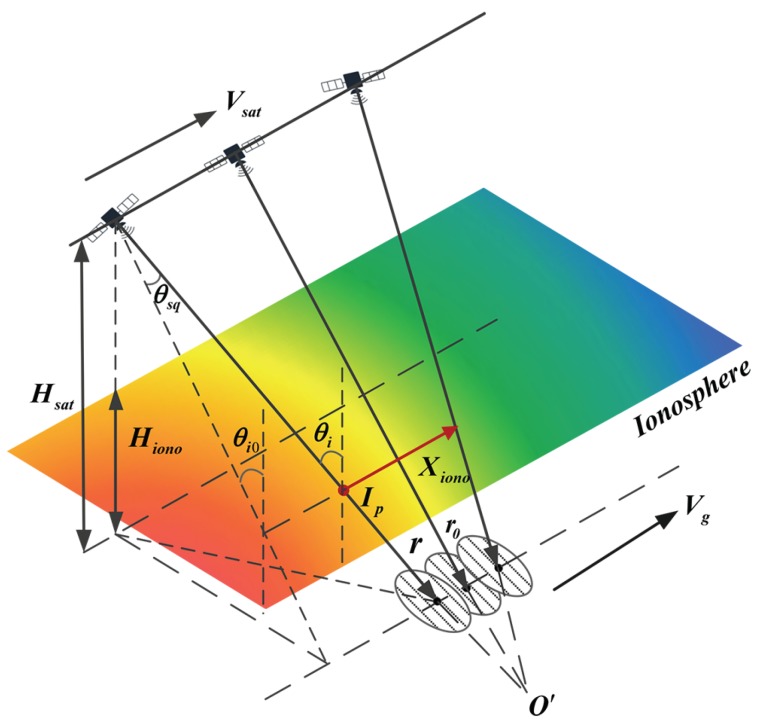
The observation geometry of sliding spotlight SAR with ionosphere.

The sliding spotlight mode SAR can make a good balance between the azimuth resolution and imaging scene by controlling the velocity of beam footprint [10,11]. The observation geometry of space-borne sliding spotlight SAR is shown in Figure 1. The beam center points at the steering point position $O^{\prime }$ within the entire acquisition time. Technically, the turbulent ionosphere can be considered to be a very thin phase screen at an equivalent altitude and the radar beam scans over the phase screen within the integration time. $H_{sat}$ is the orbit height of radar platform and $H_{iono}$ is the equivalent ionospheric height at 350 km. $I_{P}$ represents the ionospheric penetration point (IPP) and $X_{iono}$ is the IPL within acquisition time. $V_{g}$ and $V_{sat}$ represent the ground velocity and the platform velocity, respectively. $\theta_{i0}$ is the ionospheric incident angle of beam center in zeros Doppler plane. Due to the steering of antenna, the beam central incident angle $\theta_{i}$ varies within the acquisition time and is determined by the instant squint angle $\theta_{sq}\left(\eta \right)$. The relationship is given as follow

(1) $$\begin{array}{c} \theta_{i}=\arccos\left(\cos\left(\theta_{i0}\right)\cdot \cos\left(\theta_{sq}\left(\eta \right)\right)\right) \end{array}$$

**Figure 2:**
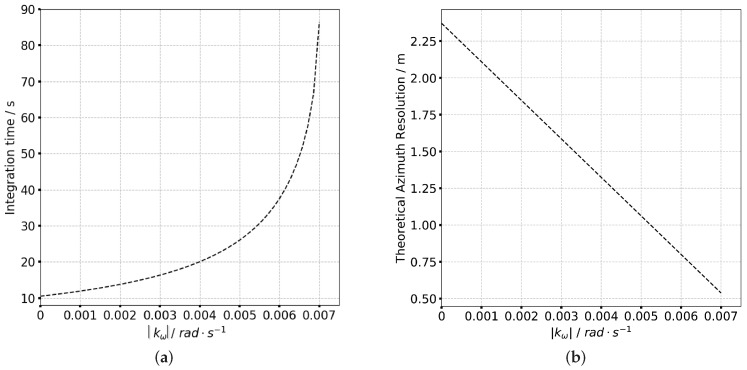
The integration time and azimuth resolution of P-band sliding spotlight mode as function of $k_{\omega }$. (**a**) Integration time. (**b**) Theoretical azimuth resolution.

We define that $R_{c}$ is the closest range between the radar platform and the scene center and $R_{rot}$ is the closest range between the radar platform and the steering point. Based on the imaging geometry, the relationship between $R_{c}$ and $R_{rot}$ is expressed as

(2) $$\begin{array}{c} \frac{R_{rot}-R_{c}}{R_{rot}}=\frac{V_{r}-\left|k_{\omega }\right|R_{c}}{V_{r}} \end{array}$$

where $V_{r}$ denotes the effective radar velocity and $k_{\omega }$ is the angular velocity of antenna steering. In particular, when $R_{rot}=+\infty$, the system is equivalent to the stripmap mode and when $R_{rot}=R_{c}$ the system is equal to the spotlight mode. The beam scanning prolongs the integration time of sliding spotlight mode which can be calculated as Ta=2Rctanλ/2D2Rctanλ/2DVgVg, where λ is the wavelength, *D* is the size of azimuth antenna and $V_{g}$ is the ground velocity which is given as follow

(3) $$\begin{array}{c} V_{g}=V_{r}-\frac{H_{sat}}{\cos\theta_{i}^{\prime }}\cdot \left|k_{\omega }\right|\cdot \sec^{2}\left(\theta_{sq}\left(\eta \right)\right) \end{array}$$

where $\theta_{i}^{\prime }$ is the ground incident angle and $\theta_{sq}\left(\eta \right)=\theta_{sq0}+k_{\omega }\eta$ is the instant squint angle. It can be seen that $k_{\omega }$ is the key factor which determines the ground velocity and the integration time. The integration time and theoretical azimuth resolution as a function of $k_{\omega }$ are shown in Figure 2. It is obvious that both the integration time and azimuth resolution significantly increase with $k_{\omega }$. When $|k_{\omega }|\;=\;0.007$ rad/s, the integration time reaches 86.49 s compared to 10.67 s for stripmap ($|k_{\omega }|\;=\;0$ rad/s), meanwhile the theoretical resolution will increase to less than 0.75 m.

As a consequence of beam scanning, the ionospheric incident angle of beam center varies within the acquisition time, whereas the traditional scintillation simulator cannot accurately simulate the beam scanning of sliding spotlight mode. Thus, in this paper, a novel scintillation simulator is proposed to accommodate the sliding spotlight geometry and exactly reconstruct the SAR raw data of sliding spotlight mode. Furthermore, a refined TFTPCF model is applied to perform the theoretical analysis for sliding spotlight mode.

## 3. Theoretical Analysis Based on TFTPCF Model

The GAF model which is first proposed by Ishimaru [19], provides a succinct model to analyze the ionospheric effect. As is shown in Figure 1, the GAF can be expressed as the coherent accumulation of the SAR signal received from the target at position r and the reference signal focus at the position $r_{0}$, which is expressed as

(4) $$\begin{array}{c} \chi \left(r,r_{0}\right)=\sum_{n}2\pi \int g_{n}\left(\omega ,r_{n}\right)\cdot f_{n}^{*}\left(\omega ,r_{0n}\right)d\omega \end{array}$$

(5) $$\begin{array}{c} \left\{\begin{array}{c} f_{n}\left(\omega ,r_{0n}\right)=u_{i}\left(\omega \right)\exp\left(j\frac{\omega }{c}2r_{0n}\right)\\ g_{n}\left(\omega ,r_{n}\right)=u_{i}\left(\omega \right)\frac{\exp\left(j2\int \beta \left(\omega \right)dl+2j\phi \left(\omega ,\rho_{n}\right)\right)}{{\left(4\pi r_{n}\right)}^{2}} \end{array}\right.\; \end{array}$$

where $f_{n}\left(\omega ,r_{0n}\right)$ and $g_{n}\left(\omega ,r_{n}\right)$ represents the reference signal and received signal at $n_{th}$ sampling point, $\psi_{iono}=2\int \beta \left(\omega \right)dl$ is the dispersive phase introduced by background ionosphere and $\phi \left(\omega ,\rho_{n}\right)$ is the scintillation phase corresponding to the IPP position $\rho_{n}$ on the scintillation phase screen and the signal frequency ω.

Based on the stop-go assumption, the SAR signal penetrates the ionospheric phase screen twice at one sampling point with different instant frequency (the SAR transmit signal is linear modulated). Thus, the random phase induced by ionospheric irregularities can be properly analyzed by the two-position two-frequency function. Based on the phase screen theory, Li et al. [20] proposed the proper TFTPCF model to study the scintillation effect from the second moment of the GAF, which is expressed as

(6) $$\begin{array}{c} \langle {\left|\chi \left(r,r_{0}\right)\right|}^{2}\rangle ={\left(2\pi \right)}^{2}\sum_{m}\sum_{n}\int_{+\infty }^{-\infty }\int_{+\infty }^{-\infty }\Gamma_{1,1}\cdot \;\exp\left(j2\left(\int_{r_{m}}k\left(\omega_{1}\right)dl\int_{r_{n}}k\left(\omega_{2}\right)dl\right)\right)\cdot \\ \;\exp\left(-j2\left(\frac{\omega_{1}r_{0m}-\omega_{2}r_{0n}}{c}\right)\right)d\omega_{1}d\omega_{2} \end{array}$$

(7) $$\begin{array}{c} \Gamma_{1,1}=\langle \exp\left\{j2\left[\phi_{1}\left(\omega_{1},\rho_{n}\right)-\phi_{2}\left(\omega_{2},\rho_{m}\right)\right]\right\}\rangle \end{array}$$

where $\rho_{m}$ and $\rho_{n}$ represent the IPP position at different azimuth position and $\langle \cdot \rangle$ is the mathematical expectation. $\Gamma_{1,1}$ is the TFTPCF which serves as a window function in the accumulation process of Equation (6). The signal decorrelation will be introduced when $\Gamma_{1,1}<0.707$ (−3 dB threshold). Based on the law of large number, the scintillation phase error tends to follow the Gaussian distribution. Thus, the Gaussian approximation can be used to simplify the TFTPCF, which is expressed as follows.

(8) $$\begin{array}{c} \Gamma_{1,1}\left(\omega_{1},\omega_{2};\rho_{n}-\rho_{m}\right)=\langle \exp\left\{j2\left[\phi_{1}\left(\omega_{1},\rho_{n}\right)-\phi_{2}\left(\omega_{2},\rho_{m}\right)\right]\right\}\rangle \\ \approx \exp\left(-2\cdot \langle {\left[\phi_{1}\left(\omega_{1},\rho_{n}\right)-\phi_{2}\left(\omega_{2},\rho_{m}\right)\right]}^{2}\rangle \right)=R_{\phi }\left(\omega_{1},\omega_{1};0\right)+R_{\phi }\left(\omega_{2},\omega_{2};0\right)-2R_{\phi }\left(\omega_{1}.\omega_{2};\Delta x\right) \end{array}$$

where $R_{\phi }\left(\omega_{n},\omega_{m};\Delta x\right)$ is the auto-correlation function (ACF) of the scintillation phase which is determined by the frequency separation ($\omega_{1}$ and $\omega_{2}$) and the 1-D IPP spatial separation ($\Delta x=∥\rho_{m}-\rho_{n}∥$). The ACF in Equation (8) is derived from the inverse Fourier transform of the power spectral density (PSD) function of the scintillation phase. In this paper, the Rino power law spectrum [29] is applied to simulate the scintillation phase screen and the ACF based on Rino’s spectrum can be expressed as

(9) $$\begin{array}{c} R_{\phi }\left(\omega_{1},\omega_{2};\Delta x\right)=r_{e}^{2}\lambda_{1}\lambda_{2}C_{s}L\sec\theta_{n}\sec\theta_{m}\cos\theta_{i}\cdot G{\left|\frac{\Delta x}{2q_{0}}\right|}^{v-1/2}\frac{K_{v-1/2}\left(q_{0}\Delta x\right)}{2\pi \cdot \Gamma_{0}\left(v+1/2\right)} \end{array}$$

(10) $$\begin{array}{c} C_{s}L=C_{k}L{\left(\frac{2\pi }{1000}\right)}^{p+1} \end{array}$$

where *G* is the gain factor, $K_{\varepsilon }\left(\cdot \right)$ is the modified Bessel function and $\Gamma_{0}\left(\cdot \right)$ is the gamma function, both $C_{s}L$ and $C_{k}L$ are the symbols of scintillation strength and p=2v is the phase spectral index, $q_{0}=2\pi /L_{0}$ is the wavenumber corresponding to the outer scales, $\theta_{n}$ and $\theta_{m}$ are the ionospheric incident angle of beam center at different sampling points.

**Figure 3:**
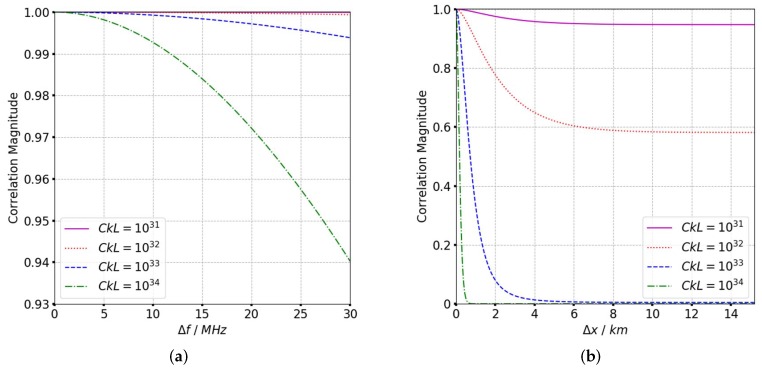
TFTPCF curves with different $C_{k}L$. (**a**) TFTPCF versus frequency separation. (**b**) TFTPCF versus spatial separation.

**Figure 4:**
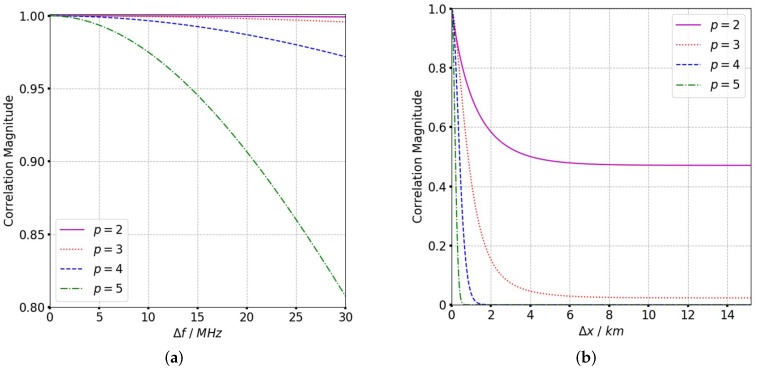
TFTPCF curves with different *p*. (**a**) TFTPCF versus frequency separation. (**b**) TFTPCF versus spatial separation.

**Figure 5:**
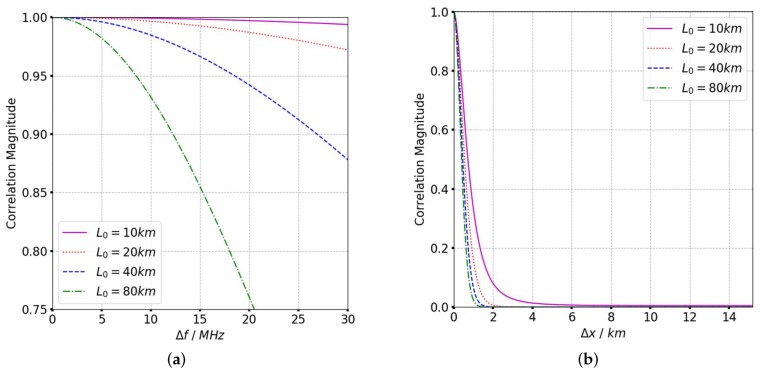
TFTPCF with different $L_{0}$. (**a**) TFTPCF versus frequency separation. (**b**) TFTPCF versus spatial separation.

In previous work, the variation of beam central incident angle is never considered due to the observation geometry of the stripmap mode. However, for sliding spotlight mode, the beam scanning leads to the increase of incident angle which will prolong the signal propagation path in irregularity layer and further aggravates the signal decorrelation. The instant ionospheric incident angle is applied as a modification of ACF for sliding spotlight mode which is presented as

(11) $$\begin{array}{c} R_{\phi }\left(\omega_{1},\omega_{2};\Delta x\right)=r_{e}^{2}\lambda_{1}\lambda_{2}C_{s}L\sec\theta_{n}\sec\theta_{sq}\left(\eta_{m}\right)\cdot G{\left|\frac{\Delta x}{2q_{L}}\right|}^{v-1/2}\frac{K_{v-1/2}\left(q_{0}\Delta x\right)}{2\pi \cdot \Gamma_{0}\left(v+1/2\right)} \end{array}$$

where $\theta_{sq}\left(\eta_{m}\right)$ is the squint angle at the $m_{th}$ sampling point. The influence of different scintillation parameters is analyzed from the TFTPCF curves. Based on the refined ACF in (11), the TFTPCF curves with different scintillation parameters are given in Figure 3, Figure 4 and Figure 5, and Table 1 presents the default value of irregularity parameters.

**Figure 6:**
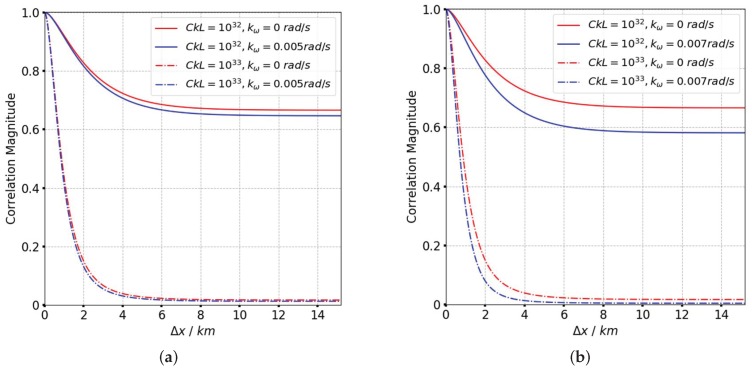
The comparison of TFTPCF curves between stripmap mode (red curves) and sliding spotlight mode (blue curves) with different $C_{k}L$ (real line: $C_{k}L=10^{32}$, dashed line: $C_{k}L=10^{33}$). (**a**) $k_{\omega }=-0.005$ rad/s (**b**) $k_{\omega }=-0.007$ rad/s.

**Figure 7:**
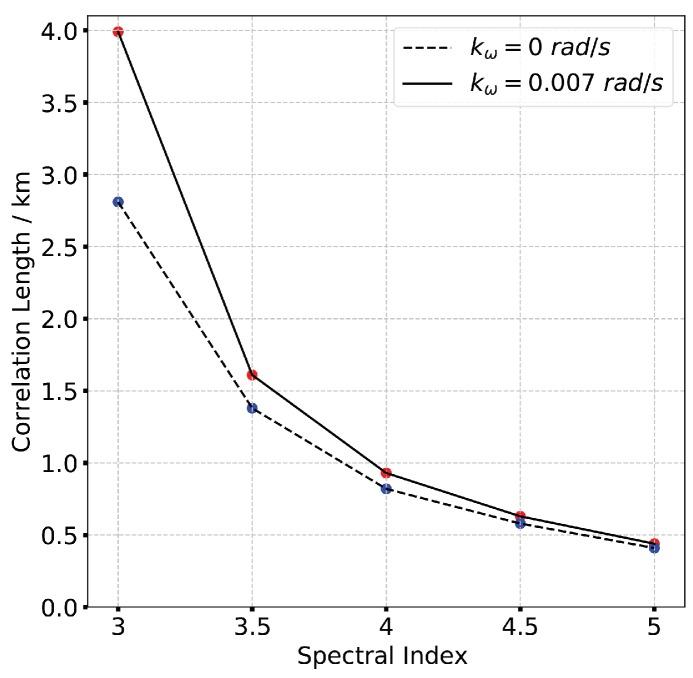
Coherent length with different spectral index.

According to Figure 3, the frequency correlation shows a significant declination with frequency separation when $C_{k}L>10^{34}$. The signal decorrelation becomes more serious with the increase of spectral index as is shown in Figure 4. In Figure 5 it is clear that the signal frequency coherence decays dramatically for $L_{0}\geq 40\;\mathrm{km}$, whereas the spatial coherence shows little difference with the increase of outer scales. For a general comparison, the signal spatial coherence is more sensitive to the scintillation strength and spectral index than outer scales. Furthermore, the signal correlation declines more significant with the increasing of spatial separation which means the spatial variation of beam central incident angle is considerable. The comparison of TFTPCF curves between the stripmap mode and sliding spotlight mode with different $C_{k}L$ is shown in Figure 6. The red curves in Figure 6 represent the TFTPCF value of stripmap mode ($k_{\omega }\;=\;0$ rad/s) and the blue curves represent the modified TFTPCF of sliding spotlight mode. It is obvious that the sliding spotlight mode is more susceptible to the scintillation effect, which also consists with the aforementioned analysis. Furthermore, the ionospheric coherent length is applied to analyze the decorrelation of P-band sliding spotlight mode, which is defined as the spatial separation Δx when $\Gamma \left(\omega_{0};\Delta x\right)\leq 0.707$. The imaging degeneration need to be considered when the IPL is longer than the ionospheric coherent length. The coherent length with different spectral index is illustrated in Figure 7, and the scintillation strength is set as $C_{k}L=10^{32}$ refers to the mildly scintillation condition.

According to Figure 7, the ionospheric correlation length dramatically declines with the increase of spectral index. The correlation length of sliding spotlight mode is less than stripmap mode, which means the sliding spotlight mode is more sensitive to the scintillation effect. The IPL of the space-borne P-band sliding spotlight SAR system is 142.03 km ($IPL=V_{sat}T_{a}$, $V_{sat}$ is the platform velocity obtained from the orbit roots). Since the IPL is significantly longer than the ionospheric coherent length, the P-band sliding spotlight mode will definitely be influenced by ionospheric irregularities. In Li’s work, the SAR resolution is defined as the absolute range separation $\delta_{r}=∥r-r_{0}∥$ by using the criterion of ambiguity function, which is expressed as

(12) χr,r02χr,r02χr0,r02=exp−2χr0,r02=exp−2

However, the redefined SAR resolution in GAF model dose not conform to the general concept of the SAR resolution based on the −3 dB criterion. Thus, the simulation of real scene is required to evaluate the scintillation effect for sliding spotlight mode quantitatively.

## 4. The ReBP-Based Scintillation Simulator for Sliding Spotlight Mode

### 4.1. Basic of Scintillation Simulator

The scintillation simulator proposed by Carrano [27] is based on the phase screen theory and has been widely acknowledged. The phase screen theory assumes that the turbulent irregularities are constrained within a very thin layer. Therefore, the ray-bending and multi-scattering effect can be neglected within the layer.

The complete SAR–SS consists of two essential steps: the phase screen simulator and propagation simulator which corresponds to the wave propagation history. When the radio wave penetrates through the ionosphere, the scintillation phase is introduced into the signal by the phase screen simulator. The 2-D scintillation phase screen is generated by multiplying the irregularity’s phase spectrum by complex white noise with unit power. After that when the radio wave transmits into the free space from the ionosphere down to the ground, the diffraction effect is simulated by the propagation simulator. It is calculated by solving the parabolic wave equation (PWE). Finally, the ionospheric transfer function (ITF) is obtained by incorporating the phase screen and propagation simulator. In this paper, a novel ReBP–based SAR–SS is proposed based on the observation geometry of sliding spotlight mode.

### 4.2. The Modified Propagation Simulator for Sliding Spotlight Mode

**Figure 8:**
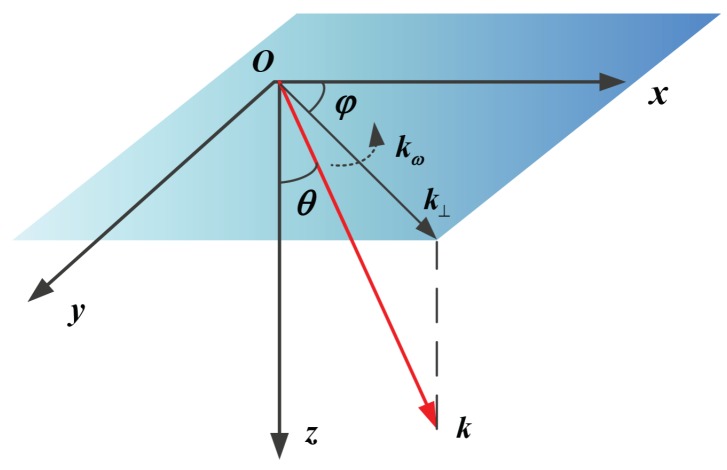
The propagation coordinate system of sliding spotlight SAR signal.

The propagation of transionospheric radio waves from the free space down to the ground follows the scalar Helmholtz equation which is expressed as

(13) $$\begin{array}{c} \nabla^{2}E\left(\rho \right)+k_{0}^{2}\left[1+\Delta \varepsilon_{r}\right]E\left(\rho \right)=0 \end{array}$$

(14) $$\begin{array}{c} E\left(\rho \right)=U\left(\rho \right)\cdot e^{jk\cdot \rho } \end{array}$$

where $k_{0}=2\pi /\lambda$ is the wavenumber corresponds to the signal frequency, $\Delta \varepsilon_{r}$ is the fluctuation term of the dielectric permittivity mainly induced by the dispersive background ionosphere, $E\left(\rho \right)$ is the electronic field, $U\left(\rho \right)$ is the complex amplitude and $\rho =\left(x,y,z\right)$ is the space vector of the electromagnetic waves defined in the geomagnetic coordinate as is shown in Figure 8. The coordinate center is chosen at the IPP position and the x-axis, y-axis, and z-axis are defined as the magnetic north, magnetic east, and vertical down to the earth. θ and φ are the ionospheric incident angle and magnetic heading of radar beam center. To make an explicit description, we neglect the inclined angle between the magnetic heading of radar platform and the magnetic east. Thus, φ is considered to be the squint angle of beam center. In Figure 8, the squint angle rotates with a constant angular velocity in acquisition time. $k=k_{0}\left(\sin\theta \cos\varphi ,\sin\theta \sin\varphi ,\cos\theta \right)$ is the transmit vector of the radio waves as well as $k_{⊥}=k_{0}\left(\cos\varphi ,\sin\varphi \right)$ is the projection of the transmit vector in horizontal plane.

By substituting the equations above and considering the Fresnel assumption (which means ∂2U∂2U∂z2∂z2≈0), the PWE in geomagnetic coordinate is expressed as

(15) $$\begin{array}{c} \nabla_{⊥}^{2}U=-k_{0}^{2}\Delta \varepsilon_{r}U+2j\frac{\partial U}{\partial x}k_{0}\sin\theta \cos\varphi +2j\frac{\partial U}{\partial y}k_{0}\sin\theta \sin\varphi +2j\frac{\partial U}{\partial z}k_{0}\cos\theta \end{array}$$

Please note that the dispersion induced by background ionosphere is not considered in the simulator, so the fluctuating part $\Delta \varepsilon_{r}$ in Equation (15) is neglected in the following derivations. Since the ionosphere irregularities are considered to be a very thin phase screen, the diffraction effect is neglected within the irregularity layer and the propagation path is considered to be a straight line for SAR signals. Based on the aforementioned assumptions, the complex amplitude of transmitted waves which has penetrated the phase screen is expressed as $U\left(\rho_{⊥},0^{+}\right)=U\left(\rho_{⊥},0\right)\cdot e^{j\phi \left(\rho_{⊥}\right)}$, where $\rho_{⊥}$ is the distance vector in the x-y plane and the $\phi \left(\rho_{⊥}\right)$ is the scintillation phase corresponds to the penetration point on phase screen. The Fourier split-step method is used to the PWE to solve the second-order derivative terms in Equation (16). Then, we derive the complex amplitude for the SAR signal as follow

(16) $$\begin{array}{c} U\left(\rho_{⊥},z\right)=U\left(\rho_{⊥},0\right)\cdot T\left(\rho_{⊥}\right) \end{array}$$

(17) $$\begin{array}{c} T\left(\rho_{⊥}\right)=F^{-1}\left\{\exp\left[j\left(\frac{\kappa^{2}\cdot z}{2k_{0}}\sec\theta \right)\right]\cdot F\left\{e^{j\phi \left(\rho_{⊥}\right)}\right\}\right\} \end{array}$$

where $\kappa =\left(\kappa_{x},\kappa_{y}\right)$ is the transverse wavenumber. The spherical wave propagation is considered in the simulator by scaling the horizontal coordinate and the propagation distance with the factor z=z1z2z1z2z1+z2z1+z2, where $z_{1}$ is the distance between the radar platform and the ionospheric height, $z_{2}$ is the ionospheric height, secθ is applied to convert the vertical distance to the oblique distance. $T\left(\rho_{⊥}\right)$ is the ITF which includes both the phase and amplitude fluctuations. The upward and downward ITF are the same since the symmetric propagation history. Therefore, the two-way ITF is calculated by squaring $T\left(\rho_{⊥}\right)$.

For sliding spotlight SAR system, the azimuthal temporal variation of beam central incident and squint angle are considered to be a modification into the original model. Here we use the penetration point at the edge of the phase screen as a reference, then the squint angle φ and incident angle θ are expressed as

(18) $$\left\{\begin{array}{c} \varphi (m)=\theta_{sq0}+k_{\omega }\frac{\Delta x_{a}\cdot m}{V_{IPP}}\\ \theta (m)=\arccos\left\{\cos\left(\theta_{i0}\right)\cdot \cos\left(\theta_{sq0}+k_{\omega }\frac{\Delta x_{a}\cdot m}{V_{IPP}}\right)\right\} \end{array}\right.$$

where Δxa=VIPPVIPPPRFPRF is the sampling distance of ionospheric phase screen at the azimuth direction and the VIPP=Re·VgRe·VgRe+Hiono·sinθiRe+Hiono·sinθi is the velocity of the IPP, where $R_{e}$ is the radius of Earth.

### 4.3. The Modified Phase Screen Simulator for Sliding Spotlight Mode

The 2-D scintillation phase screen is typically generated by applying the Gaussian noise with unit power passes through a linear filter with a specified PSD. Some research has been accomplished to study the ionospheric spectrum including the Shkarofsky spectrum, the modified Kolmogorov spectrum, and Rino power law spectrum. The Rino’s spectrum has been proved by real measured data and widely used in global ionospheric scintillation model (GISM) and wide band model (WBMOD) [24,27]. The PSD function of Rino spectrum is expressed as

(19) $$\begin{array}{c} P_{\phi }\left(\kappa \right)=\frac{r_{e}^{2}\lambda^{2}\sec^{2}\left(\theta \left(\kappa \right)\right)\cdot C_{s}L\cdot a\cdot b}{{\left[q_{0}+\left(A\kappa_{x}^{2}+B\kappa_{x}\kappa_{y}+C\kappa_{y}^{2}\right)\right]}^{\left(p+1\right)/2}} \end{array}$$

where $r_{e}$ is the classical electron radius, λ is the signal wavelength. Both *a* and *b* are structural scaling factors of irregularities along and across the magnetic field. *A*, *B* and *C* are the coefficients determined by the transmit direction and geomagnetic field whose expression has been discussed in Carrano’s work [27]. $\theta \left(\kappa \right)$ is the incident angle of beam center correlates to the spatial wavenumber. In our work, the spatial variant incident angle is applied as a modification for the original Rino spectrum and the scintillation phase screen is then derived based on the modified phase spectrum in Equation (19).

### 4.4. The Structure of ReBP-Based Scintillation Simulator

**Figure 9:**
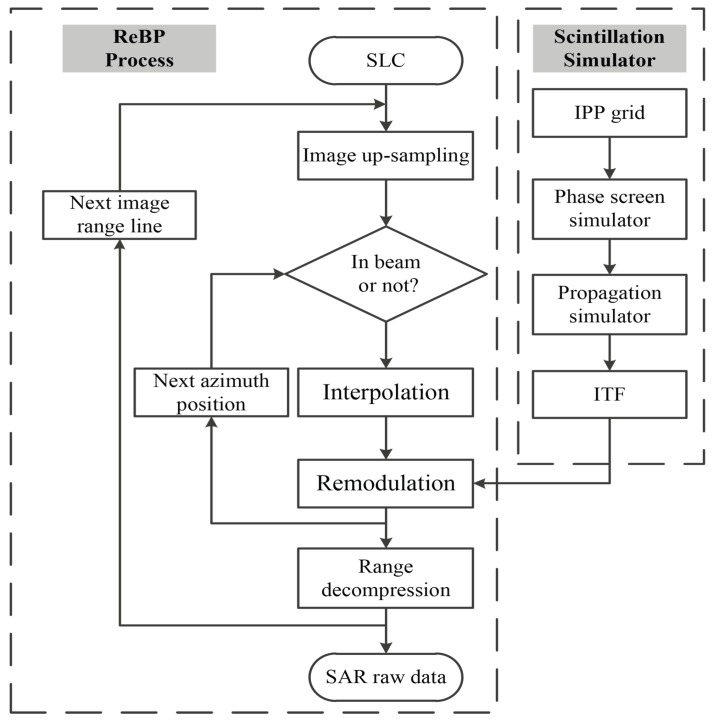
The block diagram of the ReBP–based SAR–SS.

Due to the shortage of space-borne P-band SAR data, the scintillation-contaminated SAR echo is required to be reconstructed from the SAR images. However, the existing method such as the inverse RDA cannot exactly accommodate the sliding spotlight observation geometry. The ReBP algorithm [28] provides an efficient and flexible method to simulate the SAR raw data for arbitrary imaging geometry which has been validated by the real data of Sential-1 mission. The ReBP algorithm takes the advantages of the accuracy and the expandability for analyzing the atmospheric propagation. Furthermore, the parallelization can be used in ReBP process to improve the computational efficiency. In this paper, the modified two-steps scintillation simulator is merged into the ReBP process to exactly accommodate the observation geometry of sliding spotlight mode and derive the SAR raw echo. The block diagram of the SAR-SS proposed in this paper is shown in Figure 9. According to Figure 9, the single look complex (SLC) image is given as an input and the outer loop runs for each image range line. After the up-sampling process, a projection of the azimuth beam is used to limit the illumination time of each target in the scene (shown as the SAR image pixels). In this procedure the beam scanning is considered for sliding spotlight mode. Then the interpolation is performed for the whole range line followed by the remodulation process where the ITF is introduced into the range-compressed raw data and finally after the range decompression the scintillation-contaminated raw data is acquired. By adjusting the beam projection procedure, the ReBP–based SAR–SS can also be applied to simulate the scintillation effect for TOPS and ScanSAR modes, the modifications of incident and squint angle follow the discussions in this section.

## 5. Simulation

In this section, the point target and extended target simulation are performed to present the scintillation effect on P-band sliding spotlight system, and a group of 500-time Monte-Carlo simulations are carried out to validate the theoretical analysis. The typical P-band LEO SAR system parameters are applied to carry out the simulation. The radar system and orbit parameters are shown in Table 2. The contrast simulation on stripmap mode SAR system also follows the parameters in Table 2. The simulations are performed by using the ReBP–based SAR–SS which is shown in Figure 9 and the detailed process is described as follow: The SLC image is used as the input and the image scene is defined in the earth-centered earth-fixed (ECEF) coordinate. Then the IPP grids are calculated by the positions of radar platform and scene targets and the corresponding ITFs are derived from the SAR–SS. The ReBP algorithm is used to generate the scintillation-contaminated SAR echo. Finally, the BP algorithm is used to reconstruct the SAR image from the raw data. Besides the imaging resolution, the peak power loss, peak to sidelobe ratio (PSLR) and integrated sidelobe ratio (ISLR) are considered to evaluate the imaging performance.

### 5.1. Point Target Simulation

**Figure 10:**
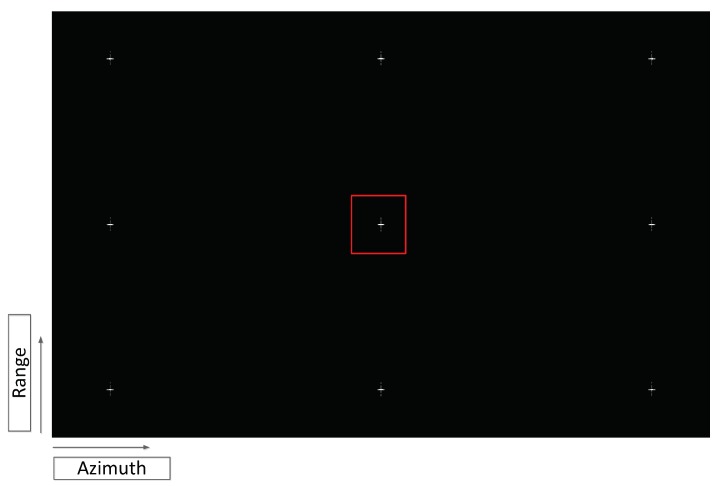
The point array target used in simulation.

**Figure 11:**
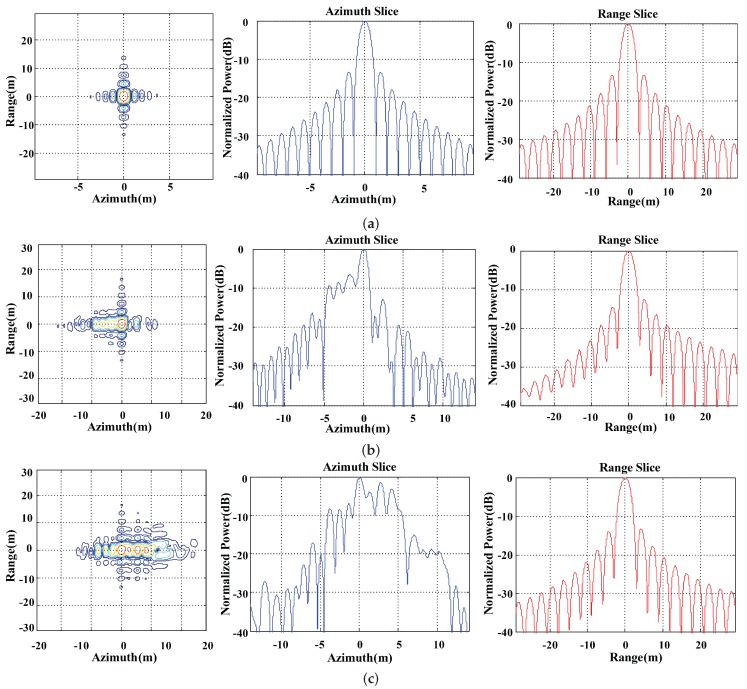
The simulation results of point targets. (**a**) Ideal imaging results. (**b**) Scintillation imaging result for $C_{k}L=10^{33}$. (**c**) Scintillation imaging result for $C_{k}L=10^{34}$.

The simulation on point targets are shown in Figure 10 and Figure 11. A 6 km × 6 km point array is applied to carry out the simulation. The origin point array is shown in Figure 10. The contour map, range and azimuth slices are shown in Figure 11. The central point target in red square is selected to perform a detail analysis. The ideal imaging result in Figure 11a has sub-meter level azimuth resolution with 0.713 m in azimuth and 2.26 m in range by using the default system parameters. The ideal image demonstrates the excellent performance of sliding spotlight mode in high-resolution SAR imaging. The scintillation strength in Figure 11b,c are $C_{k}L=10^{33}$ and $C_{k}L=10^{34}$ which refers to the moderate and strong strength of scintillation. Other ionospheric parameters are shown in Table 1 as the default value. Compared with the ideal imaging result in Figure 11a, the resolution in azimuth degenerates from 0.713 m to 4.894 m and 7.625 m in the case of $C_{k}L=10^{33}$ and $C_{k}L=10^{34}$, respectively. The more significant deteriorations are shown as the degeneration of PSLR and ISLR. According to Figure 11b, both the PSLR and ISLR decay to −3.19 dB and −3.26 dB and in Figure 11c the PSLR and ISLR drop to −1.45 dB and −0.17 dB, respectively. The extremely high PSLR and ISLR indicate that the scintillation effect will lead to serious expand of the azimuth mainlobe and further not only degenerate the azimuth resolution but also induce the peak loss. Compared with the azimuth imaging result, the distortion in range is not as serious as that in azimuth. The asymmetric sidelobe can be seen in Figure 11b mainly due to the power leakage of azimuth mainlobe.

**Figure 12:**
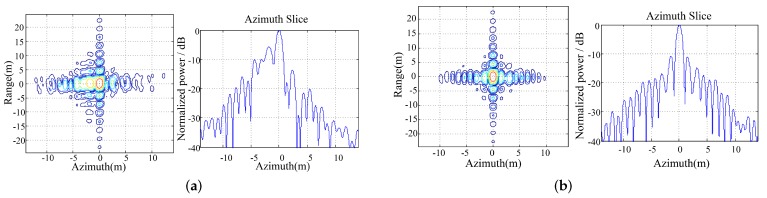
The scintillation mitigation on point target. (**a**) Scintillation imaging result for $C_{k}L=10^{33}$. (**b**) The autofocusing result.

The scintillation mitigation on point target is performed in Figure 12. The peak loss induced by ionosphere scintillation will weaken the SAR image contrast which makes the dominant scatters hard to select. Therefore, in this paper, the minimum-entropy autofocusing is applied to mitigate the scintillation effect instead of phase gradient autofocusing method. Since the spatial variation of scintillation phase screen, the autofocusing performance is limited in strong scintillation conditions. The scintillation parameters are set as $C_{k}L=10^{33}$ and p=3. The PSLR/ISLR before the autofocusing are −6.26 dB and −2.33 dB in Figure 12a. After the minimum-entropy autofocusing the PSLR/ISLR become −10.76 dB and −5.65 dB, respectively. The autofocusing result indicates that the existing scintillation mitigation method does not work perfectly even in moderate scintillation condition. As is mentioned before, the MS interferometric method [5] shed some new light on the mitigation of ionospheric scintillation, especially for sliding spotlight mode with large squint angle variations.

### 5.2. Extended Target Simulation

**Figure 13:**
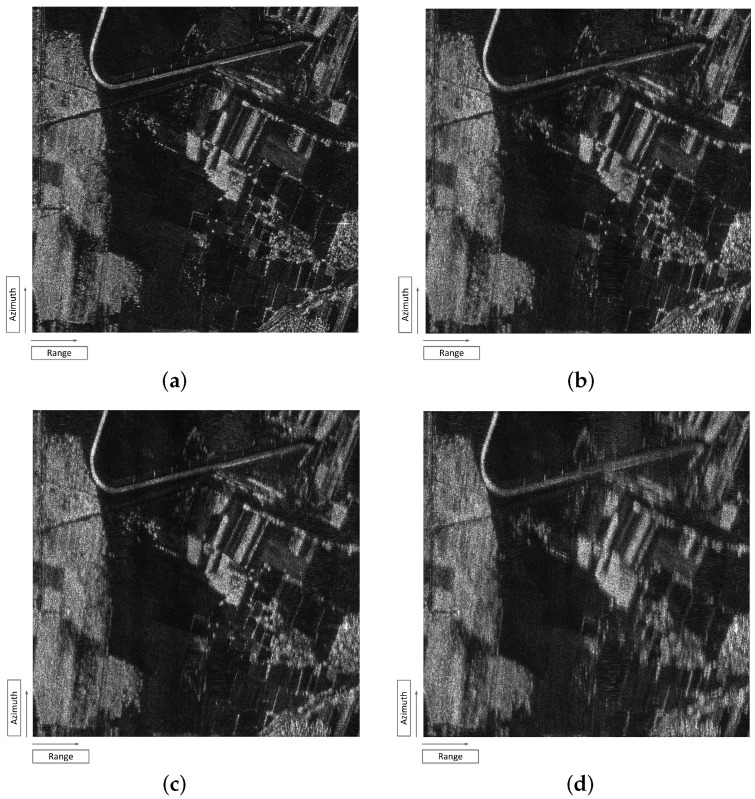
The simulation results of extended target. (**a**) Original SAR image. (**b**) Scintillation imaging result for p=3. (**c**) Scintillation imaging result for p=4. (**d**) Scintillation imaging result for p=5.

The extended target simulations are carried out by using a 2000×2000 pixels real SAR image acquired from a P-band air-borne SAR system working at 600 MHz as is shown in Figure 13a. Since the observation geometry is redefined in the beam projection and interpolation process of the ReBP algorithm, the geometry difference between two systems can be neglected in the simulation. The simulation is performed by considering the influence of different spectral index from 3 to 5, and $C_{k}L$ is set as $10^{34}$ to present a significant demonstration. Based on the theoretical analysis in Section 3, the TFTPCF serious degenerates with the increasing of spectral index. The decrease of TFTPCF will lead to the signal decorrelation and the azimuth defocusing.

**Figure 14:**
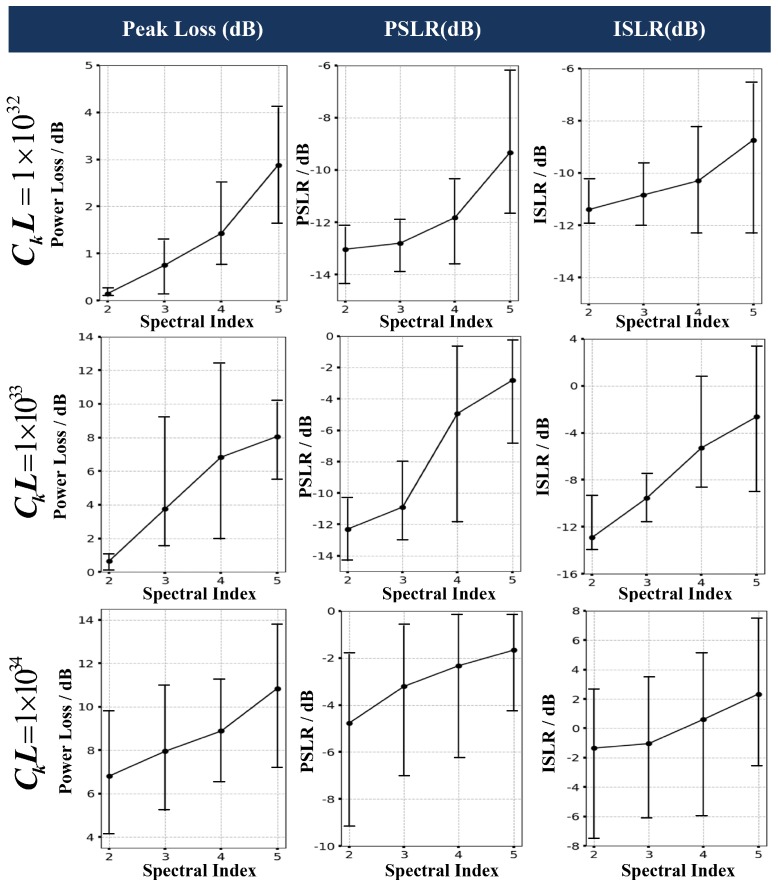
The Monte-Carlo simulation results of the scintillation effect on point targets.

Based on the quantitative analysis from the Monte-Carlo simulation listed in Figure 14, both PSLR and ISLR in azimuth increase with the rising of spectral index, which will distort the azimuth imaging performance and weaken the SAR image contrast. It can be seen from the extended target result that the image blur become more serious with the increase of spectral index. The image is still cognizable in the case of p=3. However, in Figure 13d for p=5, the scintillation-contaminated image is nearly unable to recognize. The image blur can be seen from the houses and trees in the middle of the scene. The extended target simulation corroborates the experiment result of point target that the degeneration of PSLR and ISLR induced by scintillation will seriously distort the imaging performance.

### 5.3. Monte-Carlo Simulation

As is mentioned before, the phase and amplitude scintillation is a random process. Therefore, the Monte-Carlo simulation is required to perform a statistical analysis. In our work, the simulation is iteratively performed on the point array target as is shown in Figure 10. For each group of scintillation parameters, the iterations are performed for 500 times and the statistical results are shown in Figure 14. As is discussed in Section 3, the signal decorrelation is not sensitive to the outer scale. Thus, the simulation focus on the imaging performance with different scintillation strength (from $10^{32}$ to $10^{34}$ as is shown in different rows) and different spectral index (from 2 to 5 as is shown in the x-axis of each line graph). The peak loss, PSLR and ISLR are counted and plotted as the line graph in different rows. The black spot represents the mean value of the statistical data and the vertical short lines represent the variation scope of the variables. The Monte-Carlo simulation has a good agreement with the theoretical analysis that the imaging quality degenerates with the increase of scintillation strength and spectral index. The positive ISLR happens in the case of $C_{k}L\geq 10^{33}$ and p≥4 due to the serious expand of the azimuth mainlobe. The peak loss is considerable under the scintillation, which will reduce the visibility of weak scatters and the contrast of SAR images. The experiment results also demonstrate that the scintillation effect is less serious in the case of $C_{k}L\leq 10^{32}$ and p≤2 and this can be considered to be a threshold to evaluate the influence of scintillation effect on space-borne P-band SAR system.

Another group of Monte-Carlo simulations are carried out to make a comparison between stripmap mode and sliding spotlight mode as is shown in Table 3. The simulation is performed in the case of $C_{k}L=10^{34}$, the mean value of peak loss, PSLR and ISLR are illustrated in Table 3. It is shown that all the indicators of sliding spotlight mode are lower than stripmap mode which means the scintillation effect will bring more serious distortion in sliding spotlight mode in the same ionospheric condition which validates the theoretical analysis in Section 3.

## 6. Conclusions

The space-borne P-band SAR system has a splendid prospect for its advantage in penetration ability. However, the P-band SAR imaging resolution is limited for its low central frequency and sensitivity of the ionospheric effect. In this paper, an in-depth analysis of scintillation effect is performed on P-band sliding spotlight SAR. Based on the refined TFTPCF model, the theoretical analysis indicate that the beam scanning and longer IPL will aggravate the signal decorrelation and make the sliding spotlight mode more sensitive to the ionospheric scintillation than stripmap mode. To accommodate the sliding spotlight geometry, a novel ReBP-based SAR-SS is proposed to generate the scintillation-contaminated SAR echo. The simulations on both point and extended target indicate that the scintillation-induced azimuth degeneration becomes more serious with the increasing of scintillation strength and spectral index. The Monte-Carlo simulation shows that the scintillation effect will be insignificant in the case of $C_{k}L\leq 10^{32}$ and p≤2 which can be considered to be a threshold. Since the ReBP algorithm also accommodates to TOPS and ScanSAR modes, the SAR-SS proposed in this paper can also be used to analyze the scintillation effect for these multi-mode SAR systems working in L-band or P-band. The mitigation of scintillation distortion will be further researched in the future work.

## Figures and Tables

**Table 1:** The default value of ionospheric irregularity parameters.

Parameters	Symbol	Value
Scintillation strength	$C_{k}L$	$10^{33}$
Spectral index	*p*	3
Outer scale	$L_{0}$	10 km
Irregularity structure scale	*a*/*b*	10/1

**Table 2:** Radar System and Orbit Parameters.

Parameters	Value	Unit
Carrier frequency	0.6	GHz
Bandwidth	60	MHz
Altitude of radar	700	km
Scanning angular velocity($k_{\omega }$)	−0.0055	rad/s
Semi-major Axis	7071	km
Inclination	98.6	deg
The Argument of Latitude	40	deg

**Table 3:** The comparison of scintillation effect on point targets between stripmap mode and sliding spotlight mode from Monte-Carlo simulation.

	Peak Loss/dB				PSLR/dB				ISLR/dB			
**Spectral Index**	**2**	**3**	**4**	**5**	**2**	**3**	**4**	**5**	**2**	**3**	**4**	**5**
Sliding spotlight	6.81	7.95	8.88	10.84	−4.77	−3.21	−2.33	−1.67	−1.35	−1.04	0.61	2.33
Stripmap	2.08	4.41	6.20	7.87	−6.57	−4.39	−2.76	−1.99	−3.96	−2.73	−0.45	0.36
